# Exploring the Variable Phenotypes of *RPGR* Carrier Females in Assessing Their Potential for Retinal Gene Therapy

**DOI:** 10.3390/genes9120643

**Published:** 2018-12-18

**Authors:** Anika Nanda, Anna P. Salvetti, Penny Clouston, Susan M. Downes, Robert E. MacLaren

**Affiliations:** 1 Oxford Eye Hospital, John Radcliffe Hospital, Oxford University Hospitals NHS Foundation Trust, Oxford OX3 9DU, UK; paola.anna.salvetti@gmail.com (A.P.S.); susan.downes@ouh.nhs.uk (S.M.D.); enquiries@eye.ox.ac.uk (R.E.M.); 2 Eye Clinic, Sacco Hospital, University of Milan, 20157 Milan, Italy; 3 Oxford Medical Genetics Laboratories, The Churchill Hospital, Oxford University Hospitals NHS Foundation Trust, Oxford OX3 7LE, UK; penny.clouston@ouh.nhs.uk; 4 Nuffield Department of Clinical Neurosciences, Level 6, West Wing, John Radcliffe Hospital, Oxford OX3 9DU, UK

**Keywords:** inherited retinal degeneration, retinitis pigmentosa, pigmentary retinopathy, X-linked retinitis pigmentosa, retinitis pigmentosa GTPase regulator gene (*RPGR*), female carriers, X-inactivation, skewed X-inactivation, gene therapy

## Abstract

Inherited retinal degenerations are the leading cause of blindness in the working population. X-linked retinitis pigmentosa (XLRP), caused by mutations in the Retinitis pigmentosa GTPase regulator (*RPGR*) gene is one of the more severe forms, and female carriers of *RPGR* mutations have a variable presentation. A retrospective review of twenty-three female *RPGR* carriers aged between 8 and 76 years old was carried out using fundoscopy, autofluorescence imaging (AF), blue reflectance (BR) imaging and optical coherence tomography (OCT). Confirmation of the genetic mutation was obtained from male relatives or Sanger genetic sequencing. Fundus examination and AF demonstrate phenotypic variability in *RPGR* carriers. The genetic mutation appears indeterminate of the degree of change. We found four distinct classifications based on AF images to describe *RPGR* carriers; normal (N) representing normal or near-normal AF appearance (*n* = 1, 4%); radial (R) pattern reflex without pigmentary retinopathy (*n* = 14, 61%); focal (F) pigmentary retinopathy (*n* = 5, 22%) and; male (M) phenotype (*n* = 3, 13%). The phenotypes were precisely correlated in both eyes (rs = 1.0, *p* < 0.0001). Skewed X-inactivation can result in severely affected carrier females—in some cases indistinguishable from the male pattern and these patients should be considered for *RPGR* gene therapy. In the cases of the male (M) phenotype where the X-inactivation was skewed, the pattern was similar in both eyes, suggesting that the mechanism is not truly random but may have an underlying genetic basis.

## 1. Introduction

Retinitis pigmentosa is the most-common inherited retinal dystrophy with a worldwide prevalence of 1 in 4000. It is a heterogeneous group of disorders that can be inherited as autosomal dominant (30%–40% of cases), autosomal recessive (50%–60% of cases) or X-linked (5%–15%) [1]. To date, only three genes have been identified to cause X-linked retinitis pigmentosa (XLRP) with approximately 70%–80% attributed to the Retinitis Pigmentosa GTPase Regulator (*RPGR*) gene [2].

RPGR-related RP is one of the most severe forms of retinitis pigmentosa in males, with symptoms of nyctalopia within the first 10 years of life and progression to blindness within the third or fourth decade [3]. Typical progression with the loss of rod photoreceptors, pigmentary retinopathy and perivascular changes occur with degeneration, eventually encroaching on the central macular area [4].

Despite the X-linked inheritance of *RPGR*, the severe phenotype can also be seen in female carriers [5] and is caused by the preferential inactivation of the normal X chromosome [6]. X-linked RP can, therefore, be mistakenly characterised as autosomal dominant with every generation, including females, showing typical disease characteristics [7].

In this study, we categorise the female RPGR phenotypes using fundus autofluorescence (FAF) imaging and blue reflectance imaging to define the different levels of disease. With *RPGR* gene therapy currently undergoing clinical trials for males, there must be the consideration for therapeutic intervention in females who also suffer from the severe phenotype of retinitis pigmentosa.

## 2. Materials and Methods

We reviewed 23 female carriers of X-linked Retinitis Pigmentosa who had been examined and imaged at the Oxford Eye Hospital. Patients were identified through ongoing gene therapy clinical trials for *RPGR* retinitis pigmentosa (NCT03116113) and via specialist genetic eye clinics. All females were confirmed as carriers either through male relatives screened and/or enrolled in the *RPGR* gene therapy trial (with genetic sequencing performed as per protocol) or through analysis of familial *RPGR* mutations.

All patients underwent a complete ophthalmic examination, including logMAR best-corrected visual acuity (BCVA), standardized anterior and posterior segment examination and imaging. Imaging included fundus photography (Optos, Inc., Marlborough, MA, USA), spectral domain optical coherence tomography (SD-OCT), Spectralis HRA + OCT, (Heidelberg Engineering, Heidelberg, Germany), blue autofluorescence (B-AF) and, in some patients where AF categorization was not obvious, blue reflectance (BR) (both Spectralis HRA and HRA II; Heidelberg Engineering, Heidelberg, Germany). B-AF images were obtained using a blue light excitation wavelength of 488 nm and recorded emission wavelengths were limited by a barrier filter to wavelengths between 500 and 700 nm.

## 3. Results

A total of 46 eyes of 23 consecutive RPGR patients (average age 38 years, range 8 to 76 years) were included in this study. Differences in the fundus appearance on B-AF and BR imaging allowed us to identify four different patterns of presentation: N-pattern—normal or near-normal fundus appearance, the R-pattern—radial-spoke shaped reflexes extending from the central macular area in a radial pattern; the F-pattern—focal pigmentary retinopathy patchy pigmentation with a radial reflex pattern and the M-pattern—male pattern retinitis pigmentosa. In all cases, the pattern observed in one eye matched the other in the same patient. It is possible, however, that eyes of the same patient may be functionally discordant [8] or between the boundaries of classification. These eyes can be individually categorized using the same criteria. Other similar classifications have been used to grade XLRP carriers using fundus appearance and fundus photographs [9,10].

**Figure 1:**
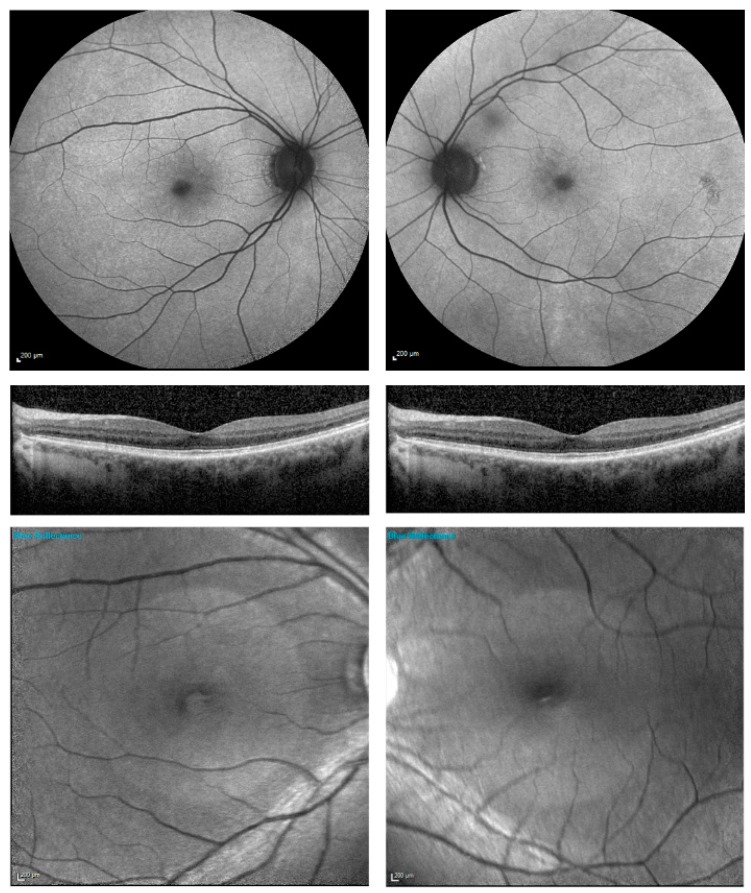
Autofluorescence imaging (AF), optical coherence tomography (OCT) and blue reflectance imaging BR of patient one classified as normal (N) pattern. There is no obvious radial pattern or pigmentary retinopathy visible on AF or 488-nm blue reflectance imaging. A radial reflex pattern may become more obvious with disease progression or with age.

The normal (N) pattern (*n* = 1) was identified in one female with an X-linked family history and confirmed *RPGR* mutation but no retinal signs or symptoms of RP, see Figure 1. The N-pattern carrier did not have any fundus abnormalities on B-AF or BR imaging, see Figure 1b. In this one case, genetic testing was carried out to confirm the carrier status of the *RPGR* mutation observed in affected males of the same family. It is possible that with the progression of the disease, a radial reflex pattern may become more obvious.

**Figure 2:**
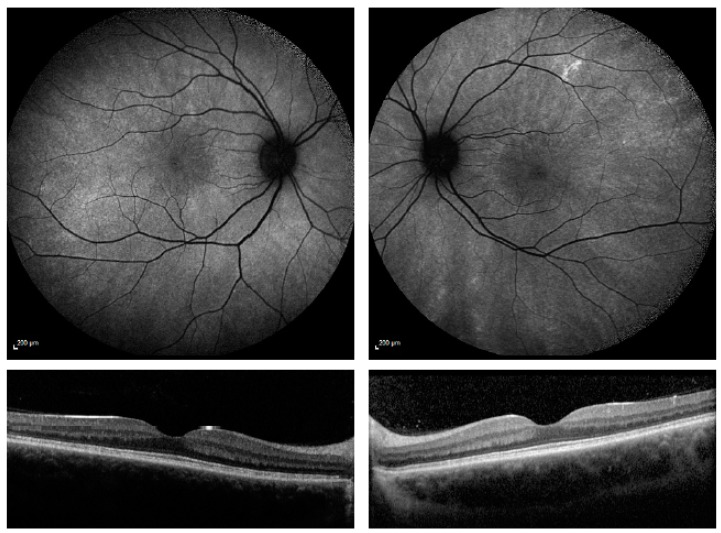
AF and OCT imaging of Radial pattern (R). A wedge-shaped reflex extends from the central retina to the periphery, sparing the macular with no pigmentary changes. The OCT shows good retinal anatomy with an intact ellipsoid layer and outer limiting membrane. Patients in this category have no symptoms of retinitis pigmentosa.

The radial (R) pattern (*n* = 14) shows a radial reflex without pigmentary retinopathy, see Figure 2. This is specific to X-linked RP female carriers. The retinal sheen creates a wedged-shaped pattern that begins at the peri-macular region and extends towards the periphery. The fovea is typically spared. Recently, cellular resolution provided by adaptive optics scanning laser ophthalmoscope at points identified with a radial reflex pattern has shown features of reduced cone density, increased inner segment diameters, and increased rod outer segment reflectivity [11].

**Figure 3:**
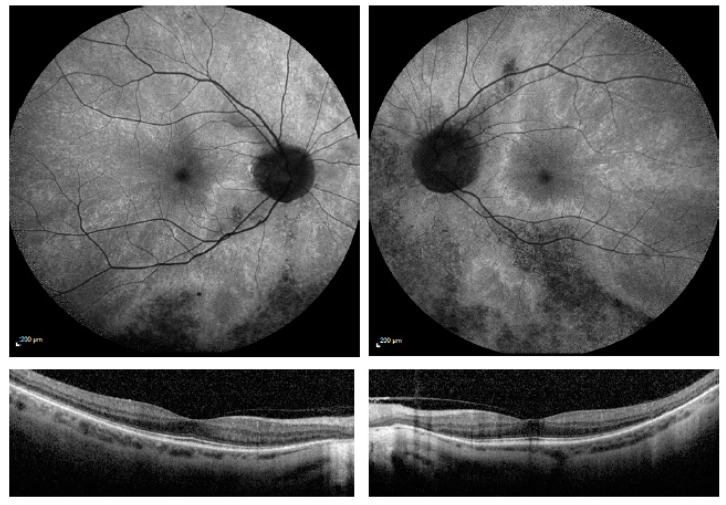
AF and OCT imaging of a Focal pigmentary pattern (F). AF images show focal pigmentary changes in the inferior retina with the radial reflex pattern sparing the macular and extending to the periphery. Area of pigmentary changes denote the areas with disrupted photoreceptors. Pigment migrates to areas devoid of photoreceptors following the pathway of the retinal vasculature. Areas of pigmentation in F-pattern appear to be random but both eyes are affected and are classified into the same category.

In the focal (F) pattern (*n* = 5), the radial pattern is still present, but with areas of focal pigmentary retinopathy, as shown in Figure 3. Similar to the pattern described in males, the areas of pigmentation in the AF corresponds to areas of degeneration of photoreceptors. Patients may complain of night blindness or visual field loss.

**Figure 4:**
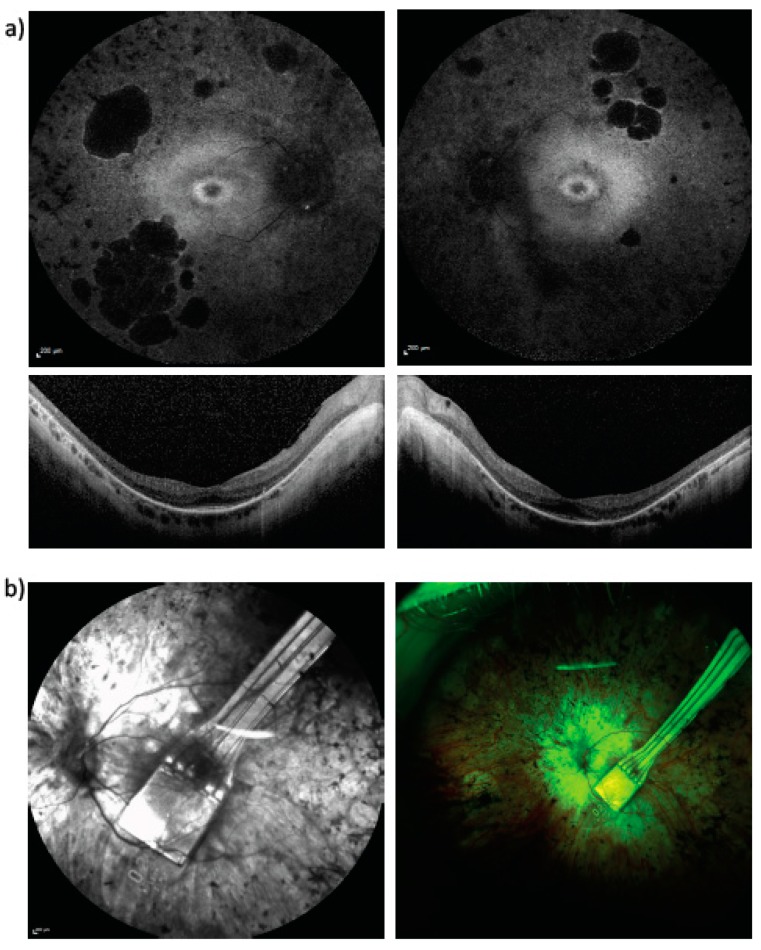

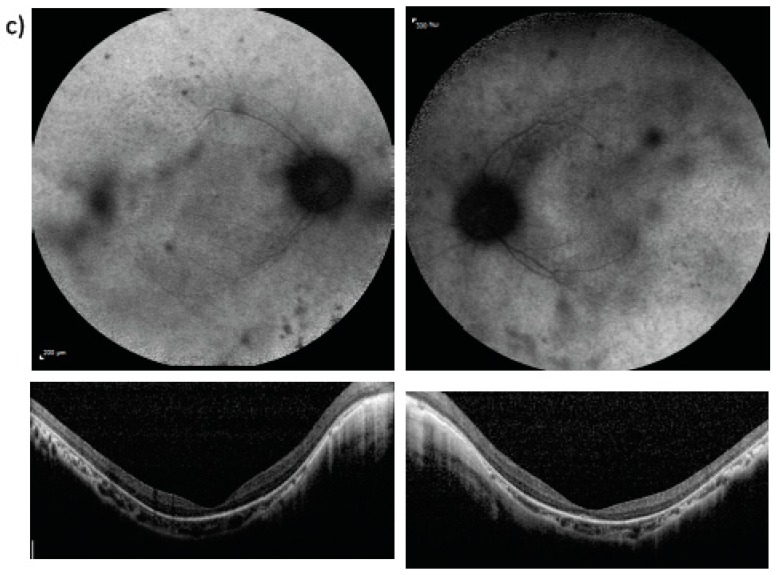
AF, OCT and Optos imaging of male pattern (M) X-linked retinitis pigmentosa (XLRP) phenotype. (**a**) (Patient 21) is a simplex case of retinitis pigmentosa GTPase regulator (*RPGR*)-associated retinitis pigmentosa with a mutation within the highly variable ORF15 region. AF imaging shows large atrophic patches with extensive peripheral pigmentary changes. A small, intact ellipsoid zone can be seen on OCT imaging, denoting the area of visual field remaining. Part (**b**) is the oldest of three patients, at 60 years old. Due to the severity of her disease she was enrolled into the retinal implant surgery trial. The AF and Optos images show the location of the implant overlying the macular with extensive pigmentary changes extending from the macular to the periphery. Part (**c**) is the youngest patient with male-phenotype retinitis pigmentosa changes and a small ellipsoid zone on OCT imaging.

**Figure 5:**
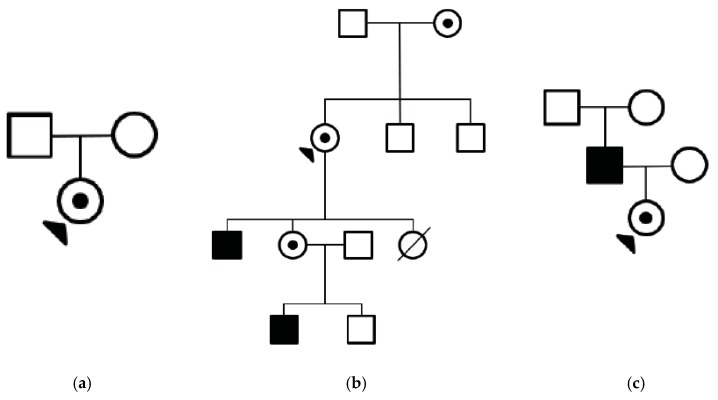
(**a**) Family pedigree of female patient 21 with Male-pattern *RPGR* retinitis pigmentosa. (**b**) Family pedigree of female patient 22 with Male-pattern *RPGR* retinitis pigmentosa. (**c**) Family pedigree of female patient 23 with Male-pattern *RPGR* retinitis pigmentosa.

Lastly, male (M) pattern (*n* = 3) represents females with the same male RP phenotype and a clinically significant visual impairment within the third or fourth decade of life, see Figure 4. This can be severe enough to cause complete sight loss, as evidenced by one female *RPGR* carrier who had sufficiently severe vision loss to be enrolled in a clinical trial for an electronic retinal implant [12]. Her son subsequently underwent *RPGR* gene therapy as part of another trial. Pedigrees for patients 21, 22, and 23 are shown in Figure 5a–c, respectively.

In all 23 cases, the pattern was the same between the two eyes, suggesting that the underlying mechanism of X-inactivation was not random but somehow genetically determined. However, different patterns were seen in a mother and daughter pair and two sisters, who would be predicted to carry a similar X-chromosome (with the exception of the small autosomal regions). There was no specific correlation between the location of the mutation and phenotype category; however, other studies such as Commander et al. have noted functional differences between mutations in the ORF15 region and exon 1–14 in *RPGR* [9]. The results from patients are summarized in Table 1. 

## 4. Conclusions

Here, we describe the spectrum of retinal degeneration in 23 female carriers of X-linked RP. In three patients (13%), we found a pattern indistinguishable to the male phenotype, verifying the previous finding that *RPGR* mutations are the third most common cause of dominant RP. In our cases, the family history of the three male pattern patients would be indistinguishable from the classic dominant inheritance [7]. Genetic testing has revealed the true identity of the disease and these cases should be considered with regard to *RPGR* gene therapy.

The *RPGR* gene is located on the short arm of the X chromosome. It is ubiquitously expressed, playing a role in cilia-related body functions including the ears and respiratory tract. RPGR^ORF15^ is the retinal-specific isoform located in the connecting cilium of photoreceptors and is thought to aid in the trafficking of molecules between the inner and outer segments [13]. The repetitive glutamic acid and glycine-rich sequence in the ORF15 region creates a mutational hotspot in the terminal exon [14].

Mutations in the *RPGR* gene are associated with a cone-rod or rod-cone phenotype. The male phenotype of XLRP is well documented, but it has long been recognised that female carriers of X-linked retinitis pigmentosa can also suffer from visual dysfunction and display a retinal phenotype even without symptoms [5]. The distinct patterns described in this cohort show the four different clinical phenotypes of *RPGR* female carriers that are thought to occur due to X-inactivation in early embryological development.

X-inactivation is a chromosomal process in females. Either the maternal or paternal X chromosome is chosen at random and is passed down to daughter cells. A 50:50 ratio of the maternal and paternal X chromosome is expected [6]. *Xist*, located on the long arm of chromosome X, is a non-translated region of RNA in the X-inactivation centre (XIC). It aids in the counting, choosing and silencing process when deciding which X chromosome should be translated [15,16]. The X-inactivation centre has both repressing and activating non-translating RNA regions that become active, dependent on the choice made—*Xist* RNA is crucially translated in the inactive chromosome that is maintained throughout the cell line [17].

Mutations may skew the inactivation process with the mutant allele being preferentially selected or inactivated, altering the ratio of mutant to wildtype X chromosomes selected. This may occur at any stage of X-inactivation and independently in the inner and outer retina due to their differing embryological origins. It is also possible that mutations in *RPGR* or adjacent genes affect the translation or selection ability of the *Xist* RNA. Peripheral blood samples in this cohort and in other studies [18] have not shown any correlation between the percentage of X-inactivation and phenotype severity. This is probably because the inactivation pattern in haemopoietic cells is different compared with the retina. Retinal biopsies are likely to be required for correlation of percentage inactivation and XLRP disease severity. Other studies have proposed alternate genetic influences to be the cause of a difference in presentation [18].

Furthermore, Table 1 demonstrates that mutation location does not correlate with a specific phenotype category. Related females 1 and 19 (sisters) have different phenotype patterns with the same mutation in *RPGR*. Additionally, patients two and three are twins and display similar phenotypes; however, twin 1 has a worse symptomatology. A long-term follow-up of patients is required to look at the progression of XLRP carrier phenotypes in more detail.

At the other end of the spectrum, carriers with the M-pattern have the most severe phenotype with similar patterns described in men and visual acuities affected early in life. Gene therapy for *RPGR*-related retinitis pigmentosa (e.g., NCT: 03116113) is currently in clinical trials for males. Additional interventional trials for severely affected females should, therefore, also be considered. Those female carriers categorised into the M-pattern are the most likely candidates for treatment; however, further formal assessments, such as visual field, scotopic microperimetry, electroretinography and assessment of stability and/or progression over time should be investigated to identify potential female candidates.

## Figures and Tables

**Table 1:** Table of results showing the age of female carrier, location of the *RPGR* mutation and the category of autofluorescence image assigned according to grading classification outlined above.

Patient Number	Current Age	*RPGR* Mutation Confirmed in Male Relative Suffering with *RPGR* Retinitis Pigmentosa	*RPGR* Exon Location	Autofluorescence Imaging Category
1	51	c.581G > A (p.Trp194Ter)	6	Normal
2	52	c.779-5T > G	8	Radial
3	26	c.904T > G	8	Radial
4	8	c.1047delT	10	Radial
5	8	c.1047delT	10	Radial
6	50	c.1377_1378	11	Radial
7	57	c.2405_2406delAG	ORF15	Radial
8	76	c.3092delA	ORF15	Radial
9	30	c.2993_2997delAAGGG	ORF15	Radial
10	55	c.2426_2427 del AG	ORF15	Radial
11	64	c.2628_2629delGG	ORF15	Radial
12	39	c.3178_3179delGA	ORF15	Radial
13	21	c.2426_2427delAG	ORF15	Radial
14	26	c.2650G > T	ORF15	Radial
15	60	c.2405_2406delAG	ORF15	Radial
16	43	c.215delA	3	Focal
17	50	c.408delT	5	Focal
18	52	c.581G > A	6	Focal
19	64	c.2426_2427delAG	ORF15	Focal
20	48	c.2993_2997delAAGGG	ORF15	Focal
21	47	c.1379T > A	11	Male
22	60	c.1571delA	13	Male
23	21	c.2405_2406delAG	ORF15	Male
